# Dilatation of the Bronchial Tubes in Children

**Published:** 1903-11-21

**Authors:** 


### DILATATION OF THE BRONCHIAL TUBES IN CHILDREN.

This is discussed in a paper by Dr. Theodore Fisher,[1] who calls especial attention to the relative frequency with which the condition is met with as a sequel of measles. Dilatation of the bronchial tubes, as seen from time to time in the course of post-mortem examinations on children, may affect both lungs throughout, or only portions of the two lungs, in which it is usually the lower lobes that are affected; or only one lung may show the lesion which may be confined to a single lobe. In those cases where the dilatation of the bronchial tubes is confined to one lung or to one lobe of a lung it may be found to have been caused by a blocking of the bronchus or one of its divisions. An enlarged caseous bronchial gland is a rare cause of such obstruction; in other cases a foreign body may be found, and Dr. Fisher thinks it possible that such a cause of dilatation of the bronchial tubes is more common than the evidence of the post-mortem room might indicate. The fact that a foreign body is not found at the necropsy does not prove that such was never present. In a case which Dr. Fisher saw both clinically and at the autopsy there was a history of a small piece of steel having entered the air passages three years before and the child's illness had dated from that event, yet at the post-mortem examination no trace of the foreign body or other cause of obstruction could be found in the bronchus of the affected lung, the bronchial tubes of which were dilated from the apex to the base. Dr. Fisher believes that the steel body may have been expectorated without attracting attention.

While, as stated above, some cases of dilatation of the bronchial tubes in children are due to pressure upon a bronchus or upon one of its divisions by a caseous gland, or to the blocking caused by a foreign body, some cases undoubtedly occur as a sequel of broncho-pneumonia. The last four instances of this that have come under Dr. Fisher's notice have apparently been occasioned by broncho-pneumonia occurring as a complication of measles. In all the cases the children were said to have been free from any symptoms of chest trouble until they were attacked with measles. Within a few weeks or months of the onset of measles death occurred, and dilatation of the bronchial tubes in varying degree was found.

[1] Lancet, Oct. 31.

